# Supplementary material for: Facile Fabrication of Transparent and Opaque Albumin Methacryloyl Gels with Highly Improved Mechanical Properties and Controlled Pore Structures
Source: Gels. 2022 Jun 10;8(6):367. doi: 10.3390/gels8060367 (PMC9222780; doi:10.3390/gels8060367)
Supplement: Supplementary file 1 [file gels-08-00367-s001.zip › gels-1722873-supplementary.pdf]

gels

Article

# Facile Fabrication of Transparent and Opaque Albumin Methacryloyl Gels with Highly Improved Mechanical Properties and Controlled Pore Structures

**Mengdie Xu†[1], Nabila Mehwish†[1] and Bae Hoon Lee [1,2]***

1. Wenzhou Institute, University of Chinese Academy of Sciences, Wenzhou 325011, China; xumd@wiucas.ac.cn (M.X.); nabila@wiucas.ac.cn (N.M.)
2. Oujiang Laboratory (Zhejiang Lab for Rengerative Medicine, Vision and Brain Health), Wenzhou 325000, China

* Correspondence: bhlee@ucas.ac.cn

† These authors contributed equally to this work.

**Supplementary Materials:**

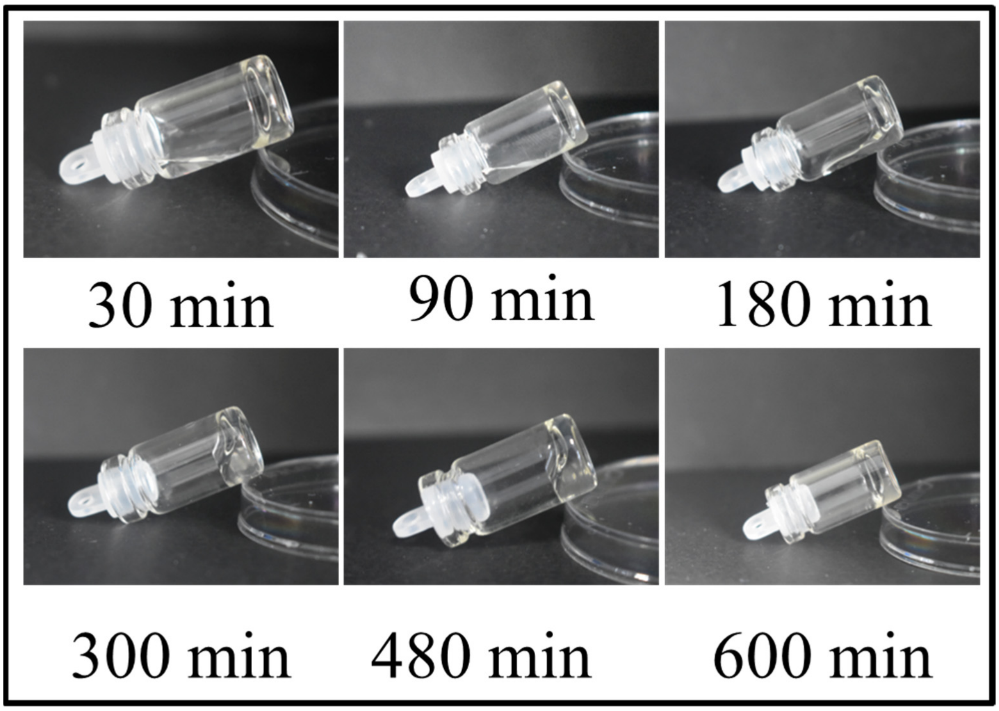


**Figure S1.** The RT sol-gel transition of 10% BSAMA as evaluated by a tilt-tube test under free radical polymerization at determined time points.

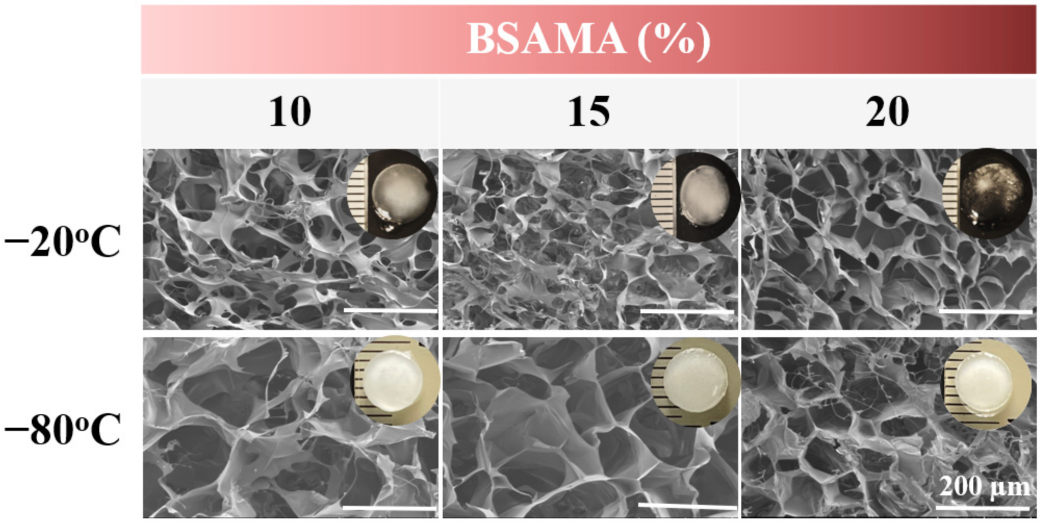


**Figure S2.** Optimization of cryogelation temperature. Representative SEM images of the gels obtained at −20 and −80 °C by using 10, 15, and 20% BSAMA concentrations. Insets in the images show the optical images of the as-obtained cryogels after thawing.

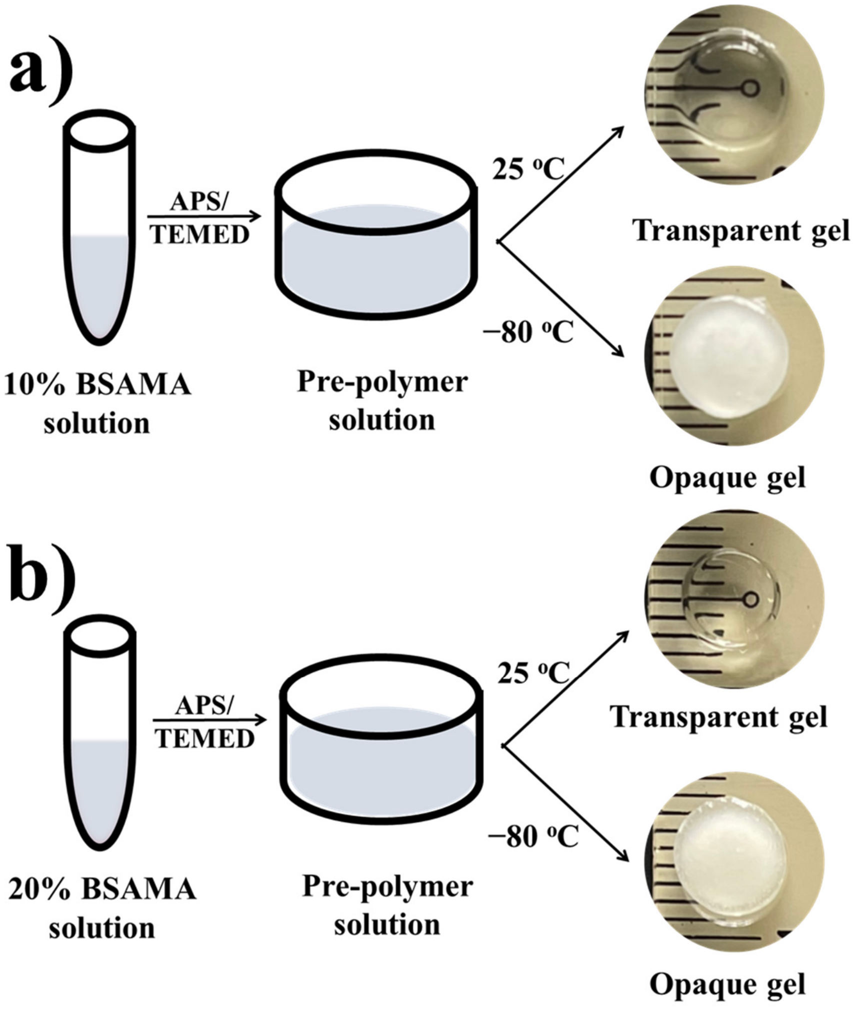


**Scheme S1.** Photographs of the transparent and opaque BSAMA gels as prepared by using 10% (a) and 20% (b) BSAMA solutions by free radical polymerization under different temperatures.

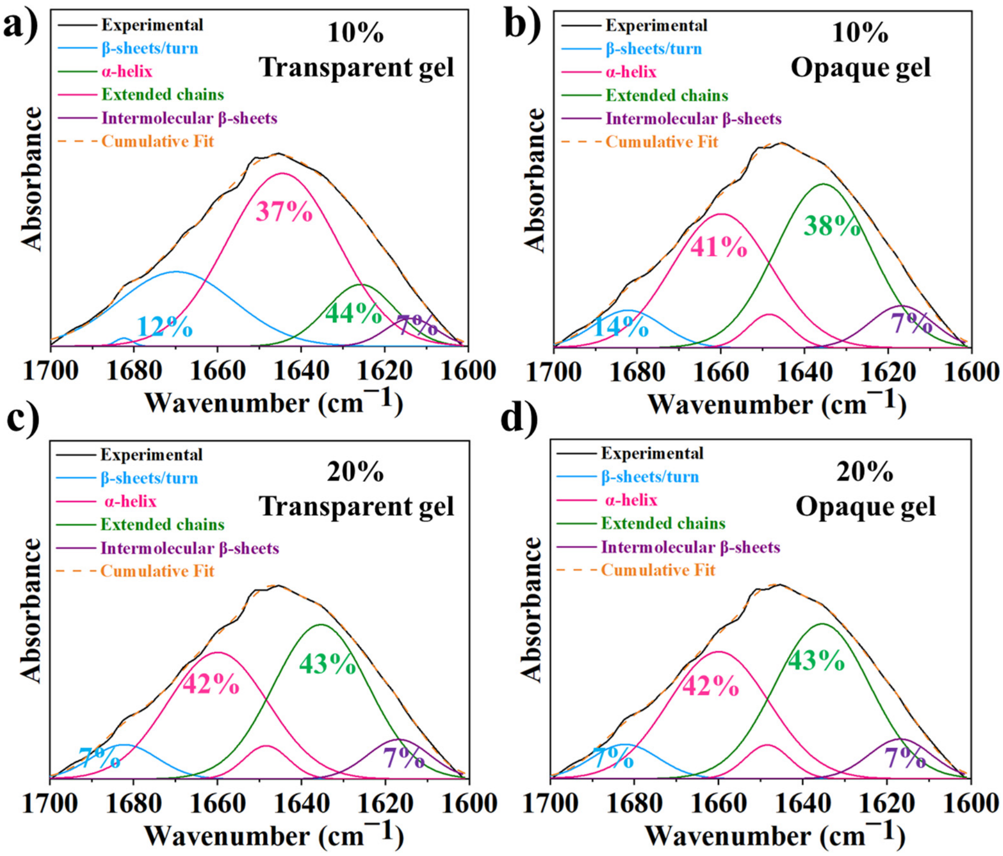


**Figure S3.** Normalized ATR-FTIR spectra of 10% BSAMA-based (**a**) transparent, (**b**) opaque gels, and 20% BSAMA based (**c**) transparent and d) opaque gels.

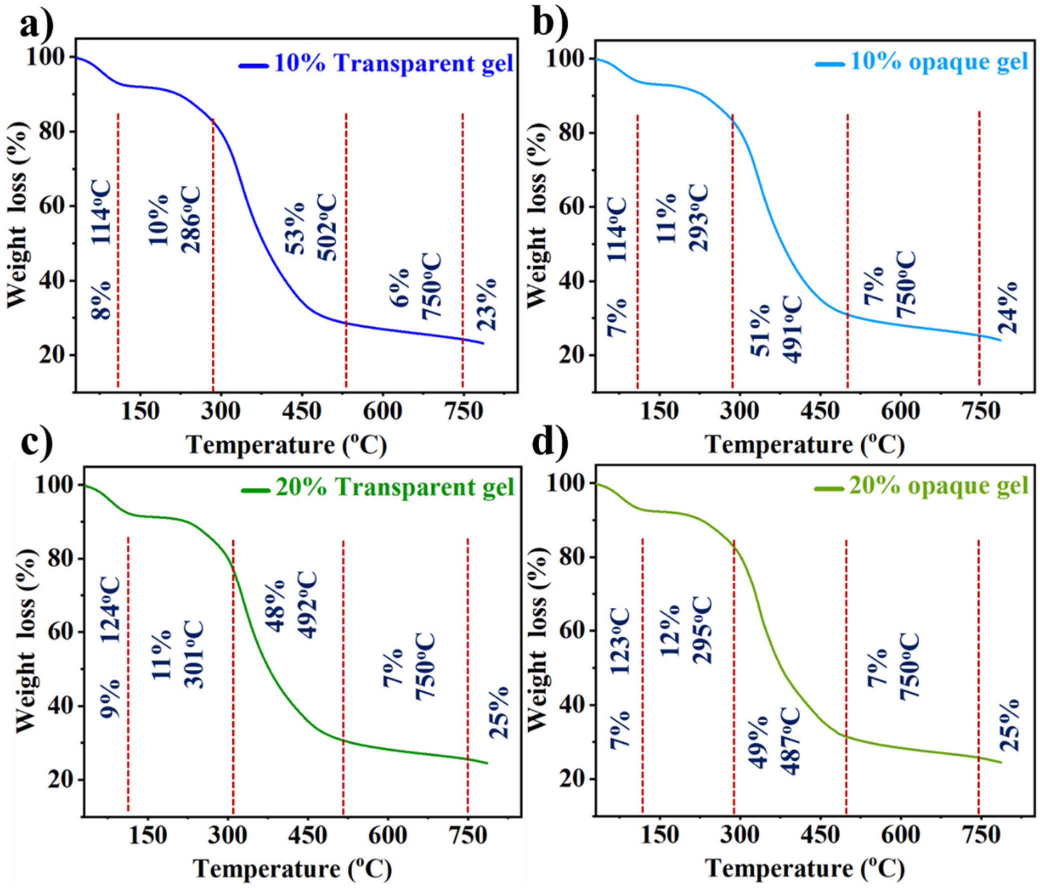


**Figure S4.** TGA curve of BSAMA (10% (w/v))-based (**a**) transparent and (**b**) opaque gels, and BSAMA (20% (w/v))-based c) transparent and d) opaque gels.

**Table S1.** Enzymatic degradation of BSAMA gels prepared at RT and −80 °C by using Proteinase K.

| 10% RT-Transparent gel | | | | | | | | |
|---|---|---|---|---|---|---|---|---|
| **Time (min)** | **Weight (mg)** | **Weight (mg) (3 replicate samples)** | | | **Residual rate (%) = $W_t/W_0$*100** | | | **Av. Residual rate (%)** |
| | | $W_1$ | $W_2$ | $W_3$ | $D_1$ | $D_2$ | $D_3$ | $= (D_1 + D_2 + D_3)/3$ |
| 0 | $W_0$ | 134.90 | 134.0 | 133.0 | 100 | 100 | 100 | 100 |
| 10 | $W_{10}$ | 126.2 | 123.6 | 128.7 | 93.55 | 92.24 | 96.77 | 94.19 |
| 20 | $W_{20}$ | 119.9 | 112.1 | 117.6 | 88.88 | 83.66 | 88.42 | 86.99 |
| 30 | $W_{30}$ | 87.8 | 88.4 | 87.7 | 65.09 | 65.97 | 65.94 | 65.67 |
| 40 | $W_{40}$ | 59.9 | 66.7 | 69.8 | 44.40 | 49.78 | 52.48 | 48.89 |
| 50 | $W_{50}$ | 35.9 | 43.3 | 45.8 | 26.61 | 32.31 | 34.44 | 31.12 |
| 60 | $W_{60}$ | 25.4 | 31.5 | 35.7 | 18.83 | 23.51 | 26.84 | 23.06 |
| 120 | $W_{120}$ | 0 | 0 | 0 | 0 | 0 | 0 | 0 |
| 180 | $W_{180}$ | | | | | | | |
| 240 | $W_{240}$ | | | | | | | |
| 300 | $W_{300}$ | | | | | | | |

| 10% −80-Opaque gel | | | | | | | | |
|---|---|---|---|---|---|---|---|---|
| **Time (min)** | **Weight (mg)** | **Weight (mg) (3 replicate samples)** | | | **Residual rate (%) = $W_t/W_0$*100** | | | **Av. Residual rate (%)** |
| | | $W_1$ | $W_2$ | $W_3$ | $D_1$ | $D_2$ | $D_3$ | $= (D_1 + D_2 + D_3)/3$ |
| 0 | $W_0$ | 129.3 | 131.6 | 130.8 | 100 | 100 | 100 | 100 |
| 10 | $W_{10}$ | 124.5 | 127.8 | 123.9 | 96.29 | 97.11 | 94.72 | 96.04 |
| 20 | $W_{20}$ | 120.4 | 123.0 | 117.9 | 93.12 | 93.46 | 90.14 | 92.24 |
| 30 | $W_{30}$ | 104.8 | 108.8 | 106.1 | 81.05 | 82.67 | 81.12 | 81.61 |
| 40 | $W_{40}$ | 96.6 | 103.9 | 103.1 | 74.71 | 78.95 | 78.82 | 77.49 |
| 50 | $W_{50}$ | 81.8 | 88.9 | 91.9 | 63.26 | 67.55 | 70.26 | 67.03 |
| 60 | $W_{60}$ | 74.1 | 71.8 | 80.7 | 57.31 | 54.56 | 61.69 | 57.85 |
| 120 | $W_{120}$ | 48.1 | 53.7 | 55.6 | 37.20 | 40.81 | 42.51 | 40.17 |
| 180 | $W_{180}$ | 32.6 | 40.1 | 34.3 | 25.21 | 30.47 | 26.22 | 27.30 |
| 240 | $W_{240}$ | 24.0 | 26.3 | 22.1 | 18.56 | 19.98 | 16.89 | 18.48 |
| 300 | $W_{300}$ | 16.8 | 16.2 | 13.1 | 12.99 | 12.31 | 10.01 | 11.77 |

| 15% RT-Transparent gel | | | | | | | | |
|---|---|---|---|---|---|---|---|---|
| **Time (min)** | **Weight (mg)** | **Weight (mg) (3 replicate samples)** | | | **Residual rate (%) = $W_t/W_0$*100** | | | **Av. Residual rate (%)** |
| | | $W_1$ | $W_2$ | $W_3$ | $D_1$ | $D_2$ | $D_3$ | $= (D_1 + D_2 + D_3)/3$ |
| 0 | $W_0$ | 90.6 | 91.9 | 89.2 | 100 | 100 | 100 | 100 |
| 10 | $W_{10}$ | 83.3 | 80.4 | 79.1 | 91.94 | 87.48 | 88.67 | 89.37 |
| 20 | $W_{20}$ | 75.3 | 72.8 | 71.1 | 83.12 | 79.21 | 79.71 | 80.68 |
| 30 | $W_{30}$ | 67.7 | 66.9 | 65.8 | 74.72 | 72.79 | 73.76 | 73.76 |
| 40 | $W_{40}$ | 62.3 | 61.3 | 60.8 | 68.76 | 66.70 | 68.16 | 67.87 |
| 50 | $W_{50}$ | 53.9 | 55.8 | 54.0 | 59.49 | 60.72 | 60.54 | 60.25 |
| 60 | $W_{60}$ | 49.1 | 50.6 | 48.3 | 54.19 | 55.06 | 54.15 | 54.46 |
| 120 | $W_{120}$ | 37.6 | 38.9 | 37.1 | 41.50 | 42.33 | 41.59 | 41.81 |
| 180 | $W_{180}$ | 29.2 | 33.9 | 30.2 | 32.23 | 36.88 | 33.85 | 34.32 |
| 240 | $W_{240}$ | 15.3 | 17.3 | 15.7 | 16.88 | 18.82 | 17.60 | 17.77 |
| 300 | $W_{300}$ | 0 | 0 | 0 | 0 | 0 | 0 | 0 |

| 15% −80-Opaque gel | | | | | | | | |
|---|---|---|---|---|---|---|---|---|
| **Time (min)** | **Weight (mg)** | **Weight (mg) (3 replicate samples)** | | | **Residual rate (%) = $W_t/W_0$*100** | | | **Av. Residual rate (%)** |

| | | $W_1$ | $W_2$ | $W_3$ | $D_1$ | $D_2$ | $D_3$ | $= (D_1 + D_2 + D_3)/3$ |
|---|---|---|---|---|---|---|---|---|
| 0 | $W_0$ | 133 | 138.5 | 137.1 | 100 | 100 | 100 | 100 |
| 10 | $W_{10}$ | 124.4 | 125.8 | 123.7 | 93.53 | 90.83 | 90.23 | 91.53 |
| 20 | $W_{20}$ | 118.1 | 119.4 | 117.5 | 88.79 | 86.21 | 85.70 | 86.90 |
| 30 | $W_{30}$ | 114.2 | 115.5 | 113.9 | 85.86 | 83.39 | 83.07 | 84.11 |
| 40 | $W_{40}$ | 106.5 | 107 | 107.3 | 80.07 | 77.25 | 78.26 | 78.53 |
| 50 | $W_{50}$ | 100.1 | 101.2 | 101.5 | 75.26 | 73.06 | 74.03 | 74.12 |
| 60 | $W_{60}$ | 98.6 | 99 | 98.2 | 74.13 | 71.48 | 71.62 | 72.41 |
| 120 | $W_{120}$ | 82.1 | 84.7 | 83.9 | 61.72 | 61.15 | 61.19 | 61.36 |
| 180 | $W_{180}$ | 74.5 | 75.6 | 77.5 | 56.01 | 54.58 | 56.52 | 55.71 |
| 240 | $W_{240}$ | 69.8 | 71.7 | 70.6 | 52.48 | 51.76 | 51.49 | 51.91 |
| 300 | $W_{300}$ | 67.5 | 66.9 | 71.5 | 50.75 | 48.30 | 52.15 | 50.40 |

**20% RT-Transparent gel**

| Time (min) | Weight (mg) | Weight (mg) (3 replicate samples) | | | Residual rate (%) = $W_t/W_0$*100 | | | Av. Residual rate (%) |
|---|---|---|---|---|---|---|---|---|
| | | $W_1$ | $W_2$ | $W_3$ | $D_1$ | $D_2$ | $D_3$ | $= (D_1 + D_2 + D_3)/3$ |
| 0 | $W_0$ | 128.3 | 127.8 | 129.7 | 100 | 100 | 100 | 100 |
| 10 | $W_{10}$ | 111.6 | 112.0 | 115.8 | 86.98 | 87.63 | 89.28 | 87.96 |
| 20 | $W_{20}$ | 108.2 | 107.7 | 108.1 | 84.33 | 84.27 | 83.34 | 83.98 |
| 30 | $W_{30}$ | 105.3 | 103.0 | 106.8 | 82.07 | 80.59 | 82.34 | 81.67 |
| 40 | $W_{40}$ | 93.9 | 94.7 | 96.6 | 73.18 | 74.10 | 74.47 | 73.92 |
| 50 | $W_{50}$ | 91.8 | 92.2 | 92.5 | 71.55 | 72.14 | 71.31 | 71.67 |
| 60 | $W_{60}$ | 87.3 | 86.6 | 85.9 | 68.04 | 67.76 | 66.22 | 67.34 |
| 120 | $W_{120}$ | 68.7 | 69.3 | 69.7 | 53.54 | 54.22 | 53.74 | 53.84 |
| 180 | $W_{180}$ | 57.4 | 59.2 | 57.3 | 44.73 | 46.32 | 44.17 | 45.08 |
| 240 | $W_{240}$ | 50.7 | 52.7 | 52.8 | 39.51 | 41.23 | 40.71 | 40.48 |
| 300 | $W_{300}$ | 42.4 | 43.6 | 42.3 | 33.04 | 34.11 | 32.61 | 33.26 |

**20% −80-Opaque gel**

| Time (min) | Weight (mg) | Weight (mg) (3 replicate samples) | | | Residual rate (%) = $W_t/W_0$*100 | | | Av. Residual rate (%) |
|---|---|---|---|---|---|---|---|---|
| | | $W_1$ | $W_2$ | $W_3$ | $D_1$ | $D_2$ | $D_3$ | $= (D_1 + D_2 + D_3)/3$ |
| 0 | $W_0$ | 128.3 | 127.8 | 129.7 | 100 | 100 | 100 | 100 |
| 10 | $W_{10}$ | 111.6 | 112.0 | 115.8 | 86.98 | 87.63 | 89.28 | 87.96 |
| 20 | $W_{20}$ | 108.2 | 107.7 | 108.1 | 84.33 | 84.27 | 83.34 | 83.98 |
| 30 | $W_{30}$ | 105.3 | 103.0 | 106.8 | 82.07 | 80.59 | 82.34 | 81.67 |
| 40 | $W_{40}$ | 93.9 | 94.7 | 96.6 | 73.18 | 74.10 | 74.47 | 73.92 |
| 50 | $W_{50}$ | 91.8 | 92.2 | 92.5 | 71.55 | 72.14 | 71.31 | 71.67 |
| 60 | $W_{60}$ | 87.3 | 86.6 | 85.9 | 68.04 | 67.76 | 66.23 | 67.34 |
| 120 | $W_{120}$ | 68.7 | 69.3 | 69.7 | 53.54 | 54.22 | 53.74 | 53.83 |
| 180 | $W_{180}$ | 57.4 | 59.2 | 57.3 | 44.73 | 46.32 | 44.18 | 45.08 |
| 240 | $W_{240}$ | 50.7 | 52.7 | 52.8 | 39.52 | 41.243 | 40.71 | 40.48 |
| 300 | $W_{300}$ | 42.4 | 43.6 | 42.3 | 33.05 | 34.12 | 32.61 | 33.26 |
